# Targeting Wnt/β-catenin signaling and its interplay with TGF-β and Notch signaling pathways for the treatment of chronic wounds

**DOI:** 10.1186/s12964-024-01623-9

**Published:** 2024-04-26

**Authors:** Dimakatso B. Gumede, Heidi Abrahamse, Nicolette N. Houreld

**Affiliations:** Laser Research Centre, Faculty of Health Sciences, University of Johannesburg, P.O. Box 17011, Doornfontein, 2028 South Africa

**Keywords:** Wound healing, Inflammation, Signaling Pathway, Wnt/β-catenin, TGF-β, Notch, Photobiomodulation

## Abstract

Wound healing is a tightly regulated process that ensures tissue repair and normal function following injury. It is modulated by activation of pathways such as the transforming growth factor-beta (TGF-β), Notch, and Wnt/β-catenin signaling pathways. Dysregulation of this process causes poor wound healing, which leads to tissue fibrosis and ulcerative wounds. The Wnt/β-catenin pathway is involved in all phases of wound healing, primarily in the proliferative phase for formation of granulation tissue. This review focuses on the role of the Wnt/β-catenin signaling pathway in wound healing, and its transcriptional regulation of target genes. The crosstalk between Wnt/β-catenin, Notch, and the TGF-β signaling pathways, as well as the deregulation of Wnt/β-catenin signaling in chronic wounds are also considered, with a special focus on diabetic ulcers. Lastly, we discuss current and prospective therapies for chronic wounds, with a primary focus on strategies that target the Wnt/β-catenin signaling pathway such as photobiomodulation for healing diabetic ulcers.

## Introduction

Wound healing is a complex cellular process that leads to tissue repair following injury. There are four overlapping phases of wound healing, starting with hemostasis which recruits platelet cells to the injured site for vasoconstriction, coagulation, and blood clot formation. This is followed by the inflammatory phase, which activates neutrophils and macrophages to clear microbes to prevent infection. The third phase is proliferation, which involves proliferation and migration of epithelial keratinocyte cells and fibroblasts to the injured site for re-epithelialization as well as formation of granulation tissue. The fourth phase involves tissue remodeling, which activates synthesis and deposition of the new extracellular matrix (ECM) by fibroblasts for wound contraction and scar formation [1]. This process requires tight regulation as dysregulation leads to the onset of chronic wounds. Chronic wounds do not progress through the healing process in a timely manner of 4–6 weeks, but prolong healing for up to 12 months and longer [2]. It has been reported that chronic wounds are a burden to the healthcare system as they are estimated to affect 10.5 million individuals in the United States of America [3]. Among the different types of chronic wounds, diabetic foot ulcers (DFUs) are estimated to affect 15% of the population in Africa and South America [4]. Studies have shown that chronic wounds fail to complete the wound healing process due to a prolonged inflammatory phase as a result of the increased recruitment of pro-inflammatory macrophages, and increased secretion of pro-inflammatory cytokines such as interleukin-1β (IL-1β) and tumor necrosis factor-α (TNF-α) at the wound site [5, 6]. Furthermore, current strategies such as wound dressing and wound debridement are reported to be less effective for treating chronic wounds [3, 7], which indicates the need for new advanced treatment modalities.

Regulation of the wound healing process is mediated by several signaling pathways, which include the transforming growth factor-beta (TGF-β), Notch, and Wnt/β-catenin signaling pathway [8]. These pathways are involved in activating the expression of target genes as well as the synthesis and secretion of soluble proteins that mediate cell activation and transition through the healing phases [8–10]. Moreover, these signaling pathways interact with one another, promoting the advancement of the wound healing process [9, 11]. Dysregulation of these signaling pathways during wound healing delays tissue repair, leading to the onset of chronic wounds. This review will discuss the role of the TGF-β, Notch, and Wnt/β-catenin signaling pathways in wound healing, with a special focus on the Wnt/β-catenin signaling pathway in the different phases of wound healing. We will then define the target genes regulated by the Wnt/β-catenin signaling pathway in the cell types involved in wound healing, namely macrophages, epithelial cells (keratinocytes), and fibroblasts. We will further discuss the crosstalk between Wnt/β-catenin signaling with the Notch and TGF-β signaling pathways during wound healing, and its modulation in chronic wounds, with the primary focus on diabetic wounds/ulcers. Lastly, we will also discuss prospective therapies for the treatment of chronic wounds which target the activation of Wnt/β-catenin signaling, with emphasis on DFUs.

### Cellular signaling in wound healing

Following injury, the hemostasis phase is activated by tissue factor (TF), a membrane glycoprotein that forms part of the clotting cascade that activates platelet cells, and monocytes upon exposure to blood [12]. TF together with damage-associated molecular pathogens (DAMPs) such as cell debris, RNA, and pathogen-associated molecular patterns (PAMPs) (e.g. bacterial lipopolysaccharides) activates the inflammatory phase which overlaps with hemostasis and initiates clot formation [13]. The blood clot fills the wound bed and forms a provisional wound matrix for the migration of leukocytes and platelet cells [6]. Platelet cells further secrete platelet-derived growth factor (PDGF) and the TGF-β1 cytokine which activate the TGF-β signaling pathway during inflammation [14].

The proliferative phase, which focuses on re-epithelialization of keratinocytes, angiogenesis, and formation of granulation tissue, is initiated by the release of cytokines (e.g. IL-4 and IL10) and growth factors such as basic fibroblast growth factor (bFGF) released by the reparative anti-inflammatory (M2) macrophages [6, 15]. Macrophages further release nitric oxide (NO) and TGF-β cytokines, which activate the proliferation and migration of fibroblast cells [16]. NO released by macrophages also activates existing endothelial cells to proliferate and secrete vascular endothelial growth factor (VEGF) for angiogenesis [17]. Cells at the edge of the wound are also activated and release EGF, keratinocyte growth factor (KGF) and insulin growth factor-1 (IGF-1), which induce the proliferation and migration of keratinocytes, endothelial cells, and fibroblasts. Mast cells, which are found in connective tissue of the skin and mucosa also secrete IgE antibodies, histamine, and cytokines such as IL-6 and IL-8 during the overlap between the inflammatory and proliferative phases [18]. They also secrete proteases such as chymase and tryptase, which breakdown the basement membrane and old ECM for the formation of granulation tissue [18]. Mast cells are further suggested to activate the proliferation of fibroblasts and endothelial cells by secreting IL-4 and VEGF during the proliferative phase [18, 19]. Activated fibroblasts begin to express alpha smooth muscle (α-SMA) and transdifferentiate into myofibroblasts for migration and deposition of ECM proteins at the wound site [20]. The provisional wound matrix is replaced by granulation tissue, which is largely composed of fibroblasts and myofibroblasts, M2 macrophages and new blood vessels to provide a scaffold for cell adhesion, migration, and cell differentiation during wound repair [6]. Keratinocytes and fibroblasts secrete matrix metalloproteinases (MMPs) such as MMP-2 and MMP-9, which are known to degrade the provisional matrix for deposition of new ECM rich in fibronectin, type I and type III collagen required for cell migration and the formation of granulation tissue [21, 22]. TGF-β signaling is active at the remodeling phase, which involves the maturation of granulation tissue where there is an increased number of myofibroblasts for ECM deposition and wound contraction [23]. At this phase, myofibroblasts and macrophages release MMPs and tissue inhibitor metalloproteinases (TIMPs) to resolve the immature ECM found in granulation tissue, and deposit increased levels of type I collagen, which has a high tensile strength [22, 24].

### The role of TGF-β and Notch signaling pathways in wound healing

The activation and role of TGF-β signaling is well-characterized in wound healing. Briefly, the binding of TGF-β ligands (TGF-β1, TGF-β2, and TGF-β3) to the TGF-β receptor I/II (TGFβRI/ TGFβRII) heterodimeric complex activates the signaling pathway. This leads to the phosphorylation of the TGF-β receptor complex, which subsequently phosphorylates receptor SMADs (R-SMADs) (SMAD2/3) proteins that bind to SMAD4 for nuclear translocation and transcriptional activation of target genes [25, 26]. Secretion of TGF-β1 also activates polarization of macrophages to the M2 phenotype, which mediates progression from the inflammatory phase to the proliferative and remodeling phases [27]. TGF-β1 also leads to epithelial-mesenchymal transition EMT in epithelial cells for re-epithelialization, as well as the transdifferentiation of fibroblasts into myofibroblasts [9]. The TGF-β/SMAD2/3 signaling pathway activates the expression of genes that encode collagens I, III and IV, as well as α-SMA, fibronectin, MMPs and tissue-inhibitors of metalloproteinases (TIMPs) in fibroblasts (Table 1) [43]. In macrophages, TGF-β/SMAD3 signaling also targets the expression of IL-10 and mediates progression from the inflammatory phase to the latter phases of wound healing [44].

**Table 1:** List of Notch and TGF-β target genes expressed during wound healing

Signaling Pathway	Wound healing phase	Target genes	Cell type	Function in wound healing	Ref.
Notch	Inflammation	*IL1B*	Macrophages	Activate switch from M1 to M2 macrophages	[28]
		*IL6*	Macrophages	Induces pro-inflammatory response	[29, 30]
		*TNF*	Macrophages	Induces pro-inflammation	[29]
	Proliferation	*ACTA2*	Endothelial cells	Endothelial-to-mesenchymal transition	[31, 32]
			Fibroblasts	Myofibroblast formation	[31]
		*VEGFA*	Endothelial cells	Angiogenesis	[33]
		*IVL*	Keratinocytes	Keratinocyte differentiation	[34]
TGF-β	Hemostasis	*TGFB1*	Platelet cells	Activate platelet aggregation, inflammatory response, and angiogenesis	[35]
	Inflammation		Macrophages	Switch from M1 to M2 macrophages	[36]
		*IL4*	Macrophages	Activates cell proliferation	[37]
	Proliferation	*COL3A1*	Fibroblasts	Collagen deposition	[16]
		*SNAI2*	Keratinocytes	Epithelial-to-mesenchymal transition	[38]
		*FN*	Fibroblasts	Deposition of new ECM	[39]
	Remodeling	*COL1A1*	Fibroblasts	Deposition of new ECM	[40]
		*ACTA2*	Fibroblasts	Myofibroblast formation	[41]
		*TGFB1*	Macrophages/Fibroblasts	Wound closure and scar formation	[42]
		*FN*	Fibroblasts	Deposition of new ECM	[40]
		*FGF2*	Fibroblasts	Wound closure	[42]

Like the TGF-β pathway, the Notch signaling pathway, is an evolutionarily conserved signaling pathway, which plays a role in embryonic development, tissue homeostasis, and tissue repair [45–47]. Notch signaling controls cell fate, proliferation, differentiation, and cell survival [48]. Notch signaling is activated by the binding of its ligands such as Jagged (JAG) 1 and − 2, and delta-like (DLL)-1, − 3, and − 4 to the Notch receptors (Notch 1–4). Upon ligand binding to the Notch receptor between adjacent cells, the Notch receptor is cleaved by γ-secretase (e.g. presenilin), leading to the translocation of the notch intracellular domain (NICD) to the nucleus where it functions as a transcription factor [49]. Transcriptional activation is mediated by binding of the NICD to recombinant binding protein-J (RBPJ) and mastermind-like (MAML) transcription co-activators, which activate the expression of genes such as the Hairy/Enhancer of Split 1 (HES1) and Hairy/E(spl)-related with YRPW (HEY), which are transcriptional repressors of the basic helix-loop-helix (bHLH) family, which regulate proliferation and differentiation of epidermal stem cells. Notch ligands are known to be highly expressed in epidermal cells, endothelial cells, keratinocytes, fibroblasts, and macrophages to activate angiogenesis and keratinocyte differentiation, and regulate inflammation [48, 50]. In the wound healing context, Notch1 signaling has been shown to activate the recruitment of M1 macrophages to the wound site and induce expression of IL-6 for angiogenesis [29, 50]. An in vivo mouse study showed that knockout of Notch1 in myeloid cells decreased macrophage recruitment and expression of TNF-α [29]. Notch activity has also been shown at the proliferative phase to induce expression of the VEGF receptor (VEGFR) in endothelial cells for angiogenesis, to activate differentiation of keratinocytes and fibroblasts, and expression of target genes required for cell migration and wound closure (Table 1) [46, 49]. Furthermore, it has been shown that inhibition of Notch signaling with N-[N-(3,5-Difluorophenacetyl)-L-alanyl]-S-phenylglycine t-butyl ester (DAPT), which inhibits γ-secretase cleavage activity, prevents fibroblast migration [46].

### Activation of the Wnt/β-catenin signaling pathway in wound healing

**Fig. 1:**
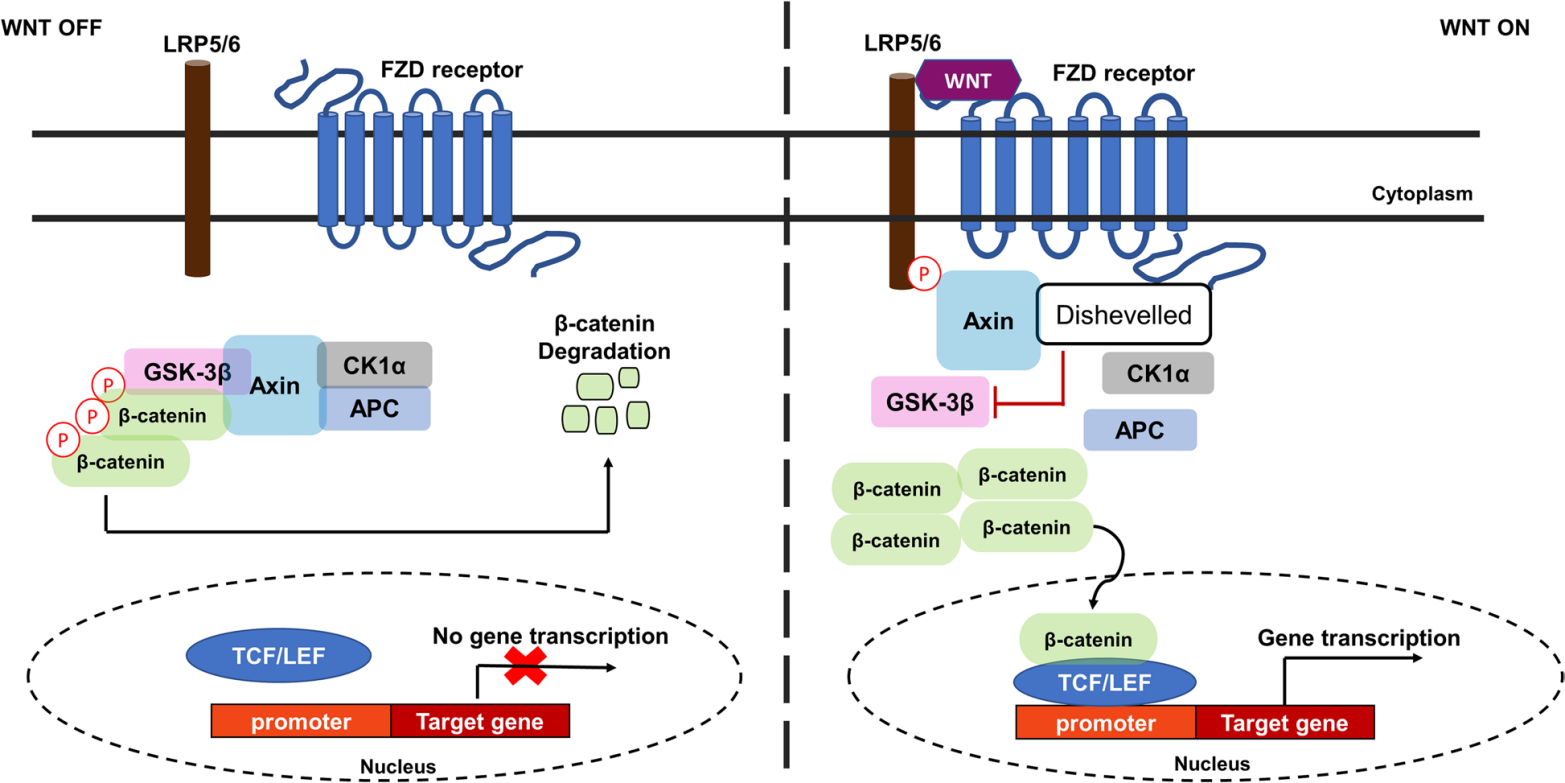
Canonical Wnt/β-catenin signaling pathway. In the absence of a Wnt ligand (WNT OFF) the “destruction complex” composed of glycogen synthase kinase-3β (GSK-3β), casein kinase 1 alpha (CK1α), Axin and adenomatous polyposis coli (APC), remains bound to β-catenin, where GSK-3β phosphorylates β-catenin which results in its degradation, thus preventing its nuclear translocation for transcription of target genes. In the presence of a Wnt ligand (WNT ON) the disheveled protein inhibits the phosphorylation of β-catenin which leads to its accumulation, stabilization, and nuclear translocation, where it binds to the T cell factor/lymphoid enhancer factor (TCF/LEF) transcription factors for transcription of target genes

The Wnt pathway is among the evolutionarily conserved pathways which plays a critical role in embryonic development, stem cell maintenance, differentiation, cell polarity, and lineage specification [51]. Nineteen Wnt genes in the human genome have been identified and indicated to activate either the Wnt/β-catenin, Wnt/planar cell polarity (Wnt/PCP), or Wnt/Ca^2+^ signaling pathway [52, 53]. Activation of these pathways requires the binding of Wnt ligands to the Frizzled (FZD) transmembrane receptor, which requires the low-density lipoproteins 5/6 (LRP5/6) receptor. The Wnt/β-catenin pathway, also known as the canonical pathway, is activated by the stabilization of β-catenin in the cytoplasm. In the Wnt inactive state (WNT OFF) (Fig. 1), β-catenin is bound to the “destruction complex” which phosphorylates β-catenin for proteasomal degradation [54]. The “destruction complex” is comprised of glycogen synthase kinase-3β (GSK-3β), adenomatous polyposis coli (APC), Axin and casein kinase 1 (CK1). The binding of Wnt ligands to FZD/LRP5/6 (WNT ON) recruits the “destruction complex” to the membrane which leads to the accumulation and stabilization of β-catenin in the cytoplasm, followed by its nuclear translocation for gene expression [51, 52]. Activation of the canonical signaling pathway by Wnt3A, for example, recruits the “destruction complex” to the cell membrane for phosphorylation by LRP5/6, leading to the dephosphorylation, accumulation and stabilization of β-catenin in the cytoplasm. Stabilized β-catenin is translocated to the nucleus for transcription of target genes (Table 2) [53, 74]. β-catenin, which is a transcriptional co-activator, binds to the T cell factor/lymphoid enhancer factor (TCF/LEF) transcription factors for the expression of target genes in specific cell types [75]**.**

**Table 2:** List of target genes activated by Wnt/β-catenin signaling during wound healing

Wound healing phase	Wnt/β-catenin target genes	Cell type	Function in wound healing	Ref.
Inflammation	*IL4*	Macrophages	Activate switch from M1 to M2 macrophages	[55, 56]
	*IL10*	Macrophages	Activate switch from M1 to M2 macrophages	[57]
	*ARG1*	Macrophages	Activates tissue repair	[55, 56]
	*EDN1*	Monocytes/macrophages	Activates cytokine production in monocytes/macrophages	[58, 59]
	*MMP9*	T cells	T cell migration to wound site	[60]
Proliferation	*CCND1*	Epithelial cells	Activates cell proliferation	[61]
	*AXIN2*	Epithelial cells	Cell proliferation	[59]
	*FN1*	Epithelial cells	Epithelial-to-mesenchymal transition	[23, 62]
	*MYC*	Epithelial cells	Promotes proliferation of epidermal stem cells and keratinocytes	[63]
	*EGFR*	Epidermal cells	Proliferation and migration of keratinocytes	[8]
	*VEGFA*	Endothelial cells	Activates angiogenesis	[8, 64]
	*FGF2*	Epithelial cells	Epithelial-to-mesenchymal transition	[65]
	*SNAI1*	Epithelial cells	Epithelial-to-mesenchymal transition	[66]
	*MMP7*	Endo/epithelial/macrophages	Formation of granulation tissue	[8, 67]
Remodeling	*COL1A1*	Epithelial cells/fibroblasts	Deposition of new ECM	[8]
	*ACTA2*	Epithelial cells/fibroblasts	Epithelial-to-mesenchymal transition	[68]
	*TIMP1*	Epithelial cells/fibroblasts	ECM deposition and scar formation	[69]
	*TGFB1*	Macrophages/Fibroblasts	Wound closure and scar formation	[70, 71]
	*CCN4*	Fibroblasts	Proliferation and migration	[72]
	*VIM*	Fibroblasts	Fibroblast to myofibroblast transition	[73]

**Fig. 2:**
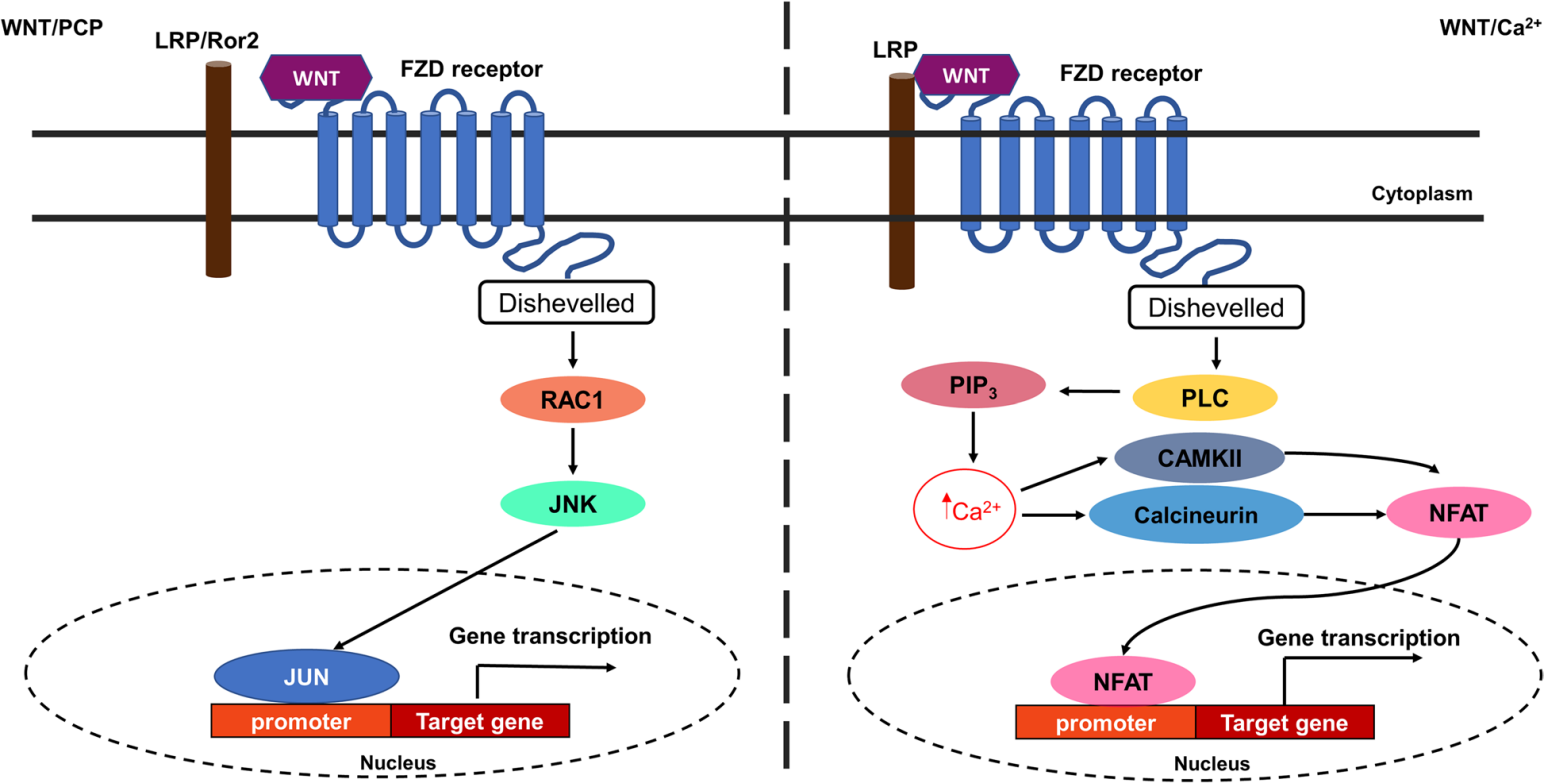
Non-canonical Wnt signaling pathways. The non-canonical Wnt/PCP pathway is involved in cell polarity and microtubule reorganization. Binding of the Wnt ligand to the FZD receptor activates the Wnt/PCP pathway either by recruiting the LRP5/6 or tyrosine kinase Ror2 receptor. This activates the GTPase RAC1 which activates the c-Jun N-terminal kinase (JNK) which leads to activation of the JUN transcription factor for expression of genes involved in cell fate and polarity. The Wnt/Ca2^+ ^activates phospholipase C (PLC) following binding of the Wnt ligand on the FZD/LRP5/6 receptor complex. Activation of PLC leads to the activation of inositol triphosphate (PIP3), which increases calcium (Ca2^+^) in the endoplasmic reticulum. Increased Ca2^+^ leads to binding interaction with calcium-calmodulin kinase II (CaMKII) and activation of the nuclear factor of activated T cells (NFAT) transcription factor for its nuclear translocation and transcriptional activation of target genes that are involved in the pro-inflammatory response

The non-canonical Wnt/PCP signaling pathway (Fig. 2) is involved in cell polarity and migration of epithelial and mesenchymal cells during development and organogenesis [76]. It is activated by Wnt4, Wnt5A, Wnt6 and Wnt11 ligands, which activate FZD independent of LRP5/6 [77]. Activation of FZD induces a signaling cascade through activation of the carboxy-terminal domain of disheveled, which activates small guanosine triphosphate (GTP) enzymes (GTPases) RHOA or RAC. Activation of RAC stimulates c-Jun N-terminal kinase (JNK) activity for cell polarity [77]. While the Wnt/PCP signaling pathway is commonly known to activate cell polarity during development and stem cell differentiation, this pathway has also been implicated in wound closure. In an in vitro wound/scratch model, treatment of embryonic mouse fibroblasts with Wnt5a, which also binds to the tyrosine Ror2 receptor, activated JNK and stimulated reorganization of the microtubules and actin cytoskeletons for “wound” closure [78]. The non-canonical Wnt/Ca^2+^ pathway is mediated by intracellular calcium (Ca^2+^). Binding of Wnt5a to the FZD/LRP5/6 receptor activates phospholipase C (PLC) and leads to increased inositol triphosphate (PIP3), 1,2 diacylglycerol (DAG), and Ca^2+^ levels [79]. Cytosolic PIP3 interacts with Ca^2+^ from the endoplasmic reticulum (ER) resulting in its release. The released Ca^2+^ interacts with calmodulin to activate calcium-calmodulin-dependent protein kinase II (CaMKII). Activated DAG can also interact with ER Ca^2+^ to activate protein kinase C (PKC). Both CaMKII and PKC can activate nuclear factor kappa-light-chain-enhancer of activated B cells (NF-кB) and nuclear factor of activated T cells (NFAT) for activation of the pro-inflammatory response and expression of several genes in different tissues (e. g. cardiac, neurons, and skeletal muscle) [79].

**Fig. 3:**
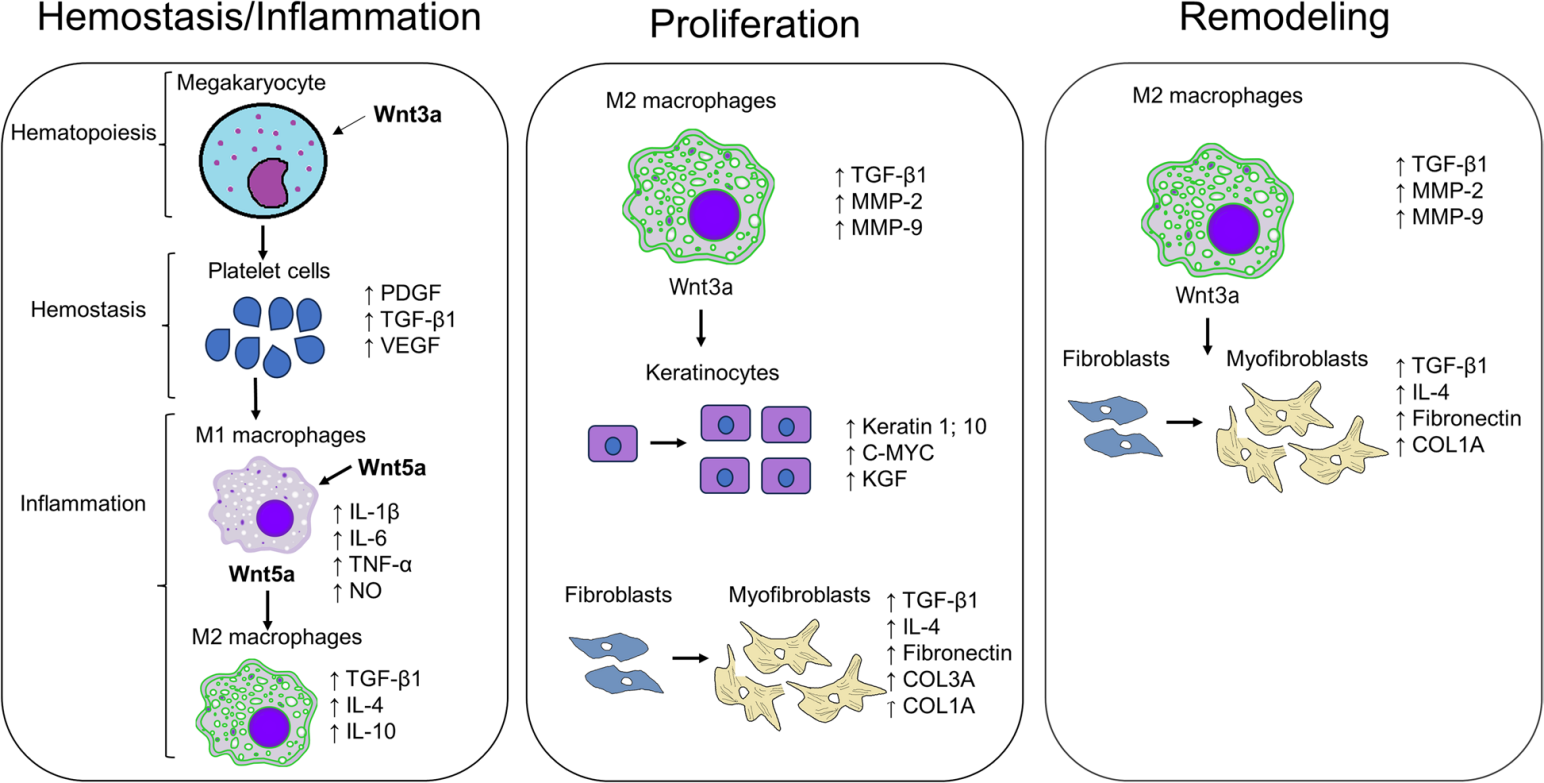
The involvement of Wnt/β-catenin signaling in wound healing. During hemostasis and inflammation, Wnt3a is known to induce the formation of proplatelet cells that mature into platelet cells and lead to the increased production and secretion of platelet-derived growth factor (PDGF), transforming growth factor-beta 1 (TGF-β1) and vascular endothelial growth factor (VEGF). The secreted growth factors from platelet cells also activate circulating and bone-marrow derived monocytes to proliferate and migrate to the wound site and differentiate into M1 macrophages which express and secrete interleukin-1β (IL-1β), IL-6, tumor necrosis factor-α (TNF-α), nitric oxide (NO), as well as Wnt5a, which further activates transdifferentiation of M1 macrophages into anti-inflammatory M2 macrophages. M2 macrophages produce and secrete anti-inflammatory cytokines (TGF-β1; IL-4; IL-10) that leading to the progression to the proliferative phase, where the TGF-β1 and Wnt3a secreted by M2 macrophages activate the proliferation and migration of keratinocytes and fibroblasts for formation of granulation tissue. M2 macrophages also secrete metalloproteinase-2 (MMP-2) and MMP-9 for degradation of old ECM and deposition of new ECM. At the proliferative phase fibroblasts also transdifferentiate into contractile myofibroblasts expressing alpha-smooth muscle actin (α-SMA), fibronectin as well as collagen type 3A1 (COL3A1) and (COL1A1). At the remodeling phase, the activated M2 macrophages and myofibroblasts further secrete TGF-β1 and Wnt3a for further proliferation and differentiation of fibroblasts into myofibroblasts for wound contraction and deposition of COL1A which is involved in scar formation

The Wnt/β-catenin signaling pathway has been shown to be involved in hematopoiesis, which is activated concurrently with the hemostasis phase upon injury (Fig. 3). During hemostasis, activation of Wnt/β-catenin signaling mediates the proliferation of megakaryocytes and proplatelet formation. A study by Macaulay et al. [80] showed that stimulation of mouse megakaryocytes by Wnt3A increased their proliferation, while treatment with Dockkopf-1 (DKK-1), a Wnt inhibitor which binds the LRP/5/6 receptor, inhibited their proliferation. Wnt signaling also induces the binding of β-catenin to the TCF4 transcription factor in macrophages, Which increases the expression of Arginase-1 (*Arg-1*) and mannose receptor (MR) required for metabolic changes that are involved in macrophage polarization from the M1 (pro-inflammatory) to the M2 (anti-inflammatory) phenotype during wound healing [81, 82]. It has also been shown that the expression and secretion of Wnt5A by macrophages at the inflammatory phase stimulates the synthesis and secretion of pro-inflammatory cytokines such as interferon-gamma (IFN-γ), IL-1, IL6 and TNF-α for clearing of infectious microorganisms [83]. Also, a separate study showed that monocyte-derived dendritic cells express high levels of Wnt5A during inflammation, leading to the secretion of the anti-inflammatory IL-10 cytokine [84]. These findings suggest that Wnt5A induces both pro-inflammatory and anti-inflammatory responses in different immune cells at the inflammatory phase.

Activation of the Wnt/β-catenin pathway at the proliferative phase leads to β-catenin binding to the TCF7L2 transcription factor for expression of target genes such as cyclin D1, matrix metalloproteinase-7 (MMP-7) which are required for cell division, and EMT in keratinocytes [26, 85]. The β-catenin/TCF transcriptional activity in macrophages is also crucial during the proliferative phase to promote granulation tissue formation and activation of macrophages for the recruitment of fibroblast cells [86]. β-catenin also induces transcription of ECM genes, which have, interestingly been shown to induce a feedback loop to regulate β-catenin [23]. β-catenin has also been shown to further activate myofibroblast formation at the remodeling phase for wound contraction and scar formation [86]. The Wnt/β-catenin signaling pathway at the remodeling phase is also reported to have disparate roles where it inhibits the migration of keratinocytes and promotes the proliferation and migration of fibroblasts [62, 87]. The regulation of Wnt/β-catenin signaling at this stage is reported to be critical as the prolonged stability of β-catenin delays wound contraction and wound size, thus increasing the risk of chronic wound healing. It is known that Wnt/β-catenin signaling activates epithelial stem cells as well as melanocyte stem cells in the hair follicle bulge for hair growth and pigmentation [88, 89]. A study by Itoh et al. [90] showed in a murine model that at Wnt/β-catenin signaling at the remodeling phase, stimulates wound-induced hair follicle formation that is similar to embryonic follicle formation as indicated by increased expression of alkaline phosphatase (*Alp*), Keratin 17 (*Krt17*), *Lef1* and *Wnt10b*. Another study by Lee et al. [91] showed in vitro that treatment of human dermal papilla cells with valproic acid (GSK3β inhibitor) activates Wnt/β-catenin signaling resulting in high expression of ALP, which indicates activation of the anagen (growth) phase of hair follicle formation. These studies thus indicate that Wnt/β-catenin is also involved in tissue remodeling.

### Crosstalk of Wnt/β-catenin with Notch and TGF-β signaling pathways during wound healing

While the TCF/LEF transcription factors are well-studied binding partners of β-catenin, several other transcription factors have been shown to bind β-catenin. These include Twist, a basic helix-loop-helix transcription factor that is involved in the EMT process by downregulating E-cadherin and N-cadherin expression levels in epithelial cells [92]; Forkhead box (FOX) transcription factor that competes with TCF/LEF for promoter occupancy on target genes for stem cell renewal and differentiation [93]; myoblast determination protein 1 (MyoD) for skeletal muscle formation and regeneration [94, 95]; and the Yes-associated protein (YAP) for tissue repair in enterocytes [96]. The binding of β-catenin with other transcription factors is associated with activation of other signaling pathways that have been suggested to crosstalk with the Wnt/β-catenin pathway. Other signaling pathways that have been shown to crosstalk with Wnt/β-catenin during wound healing include the TGF-β, Notch, and Sonic/Hedgehog (SSH) signaling pathways [8]. Furthermore, a few studies have shown some interplay between the TGF-β, Wnt/β-catenin and Notch signaling pathways during tissue repair however, this interplay has not been fully explored. This review will focus on the crosstalk between Wnt/β-catenin signaling pathways with TGF-β and Notch signaling pathways for wound healing.

### Wnt/β-catenin interacts with TGF-β signaling pathway during wound healing

The crosstalk between TGF-β and Wnt/β-catenin has been widely reported, however, the exact molecular cascade that leads to this interaction has not been fully described. Studies have suggested that the crosstalk between the TGF-β and Wnt/β-catenin signaling pathways occurs via the SMAD proteins (SMAD2/3) [73]. A study by Charbonney et al. [97] showed that treatment with TGF-β induced epithelial-myofibroblast transition by increasing α-SMA levels in the presence of β-catenin, however, knockdown of β-catenin prevented the epithelial-to myofibroblast-transition (EMyT) switch in tubular epithelial cells. Furthermore, this study showed that β-catenin-dependent expression of α-SMA was activated in a TCF/LEF-independent manner [62]. Another in vitro study showed that treatment of mouse fibroblasts with Wnt3a increased α-SMA expression to induce fibroblast-myofibroblast differentiation via increased TGF-β expression and SMAD2 phosphorylation [98]. It has also been shown that β-catenin activity mediates the expression of collagen I and IV as well as fibronectin during the proliferative phase in a wound healing mouse model [23].

**Fig. 4:**
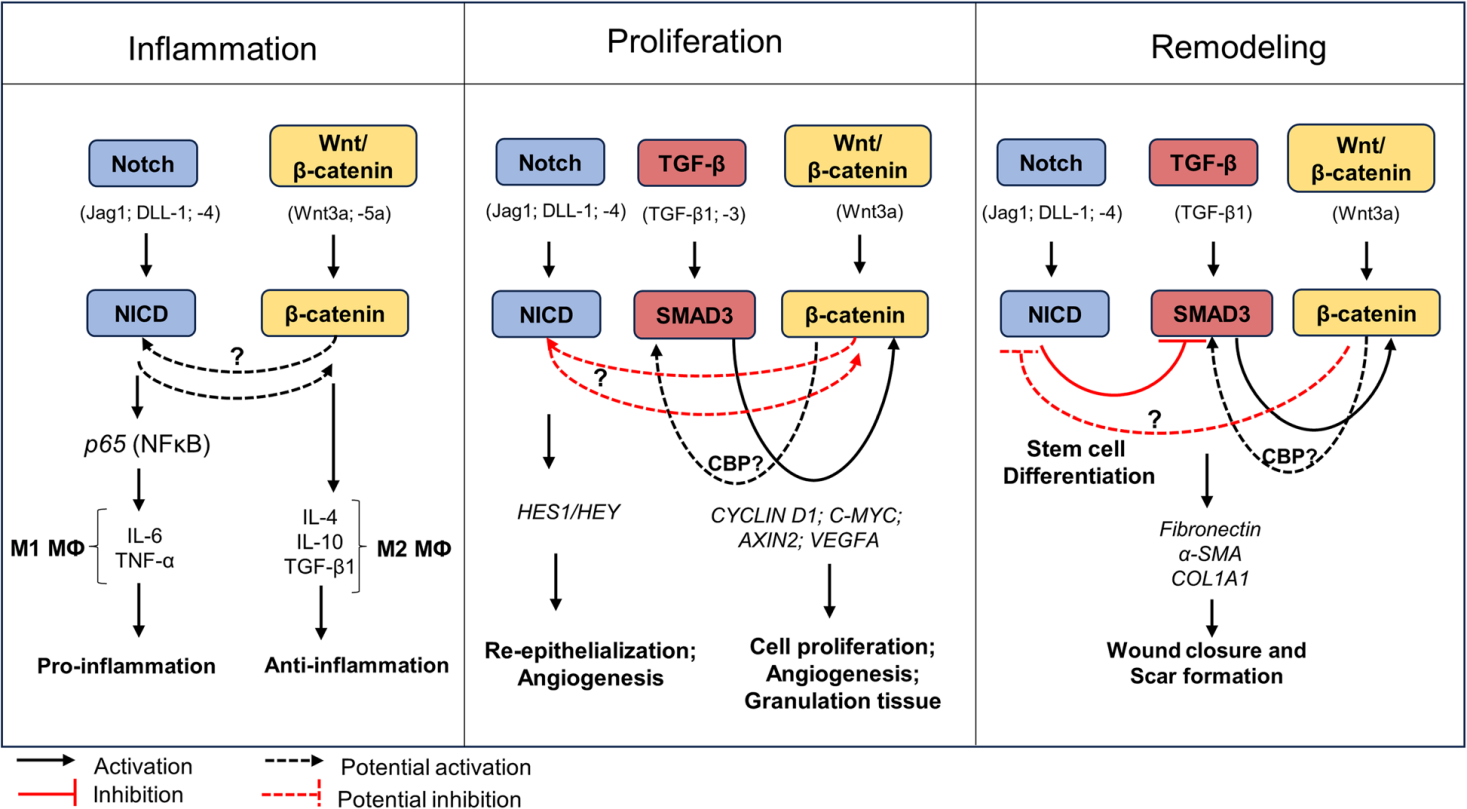
Crosstalk between Wnt/β-catenin, Notch, and TGF-β signaling pathways during wound healing. During the inflammatory response, the Notch signaling pathway is activated and interacts with necrosis factor kappa B (NFκB) to induce production of interleukin-6 (IL-6) and tumor necrosis factor-α (TNF-α). The crosstalk between β-catenin and Notch has been reported from the onset of injury. However, it is unknown if this crosstalk is activated at the start of the inflammatory phase or start of the proliferative phase. Nevertheless, this crosstalk has been shown to activate expression of *C-MYC* and *HES1*, which are both expressed at the overlap of the inflammatory phase and proliferative phase. At the proliferative phase all three of these pathways are activated, possibly in a temporal manner. It has not been indicated if NICD directly binds to β-catenin for the crosstalk between these pathways. We hypothesize that there is temporal crosstalk between Notch (NICD) and β-catenin as transcriptional co-activators at the proliferative phase. Β-catenin is known to interact with SMAD3 however it is uncertain if this interaction occurs via CBP/p300 at both the proliferative and remodeling phases. Notch signaling has also been indicated to negatively regulate Wnt/β-catenin signaling during osteogenic proliferation, while Wnt/β-catenin inhibits Notch signaling during osteogenic differentiation. This interplay has however, not been shown in cutaneous wound repair. We hypothesize that this interplay is also involved in cutaneous wound repair in a temporal manner, where β-catenin inhibits Notch activity during tissue remodeling for the trans differentiation of fibroblasts to myofibroblasts as well as for formation of the new ECM for scar formation and wound closure

There are conflicting theories regarding the crosstalk between TGF-β and Wnt/β-catenin signaling during wound healing. One of these theories suggests that activation of Wnt/β-catenin signaling leads to the expression of TGF-β, which induces SMAD2 phosphorylation, leading to the expression of target genes (e. g. *ACTA2*, and *COL1A1*) involved in wound healing [98]. Another study suggests that the presence of TGF-β leads to the activation of β-catenin [99]. The third theory suggests that the activation of SMAD3 or β-catenin activates the cyclic AMP-responsive-element-binding protein (CREB)-binding protein (CBP), which mediates SMAD3/β-catenin complex formation for expression of target genes such as α-SMA (Fig. 4) [25, 100]. It is unclear, however, if CBP mediates SMAD3/β-catenin complex formation for nuclear localization as well as transcriptional activation. CBP, which is closely related to p300, is an acetyltransferase protein and can bind to the C-terminus of β-catenin and acetylates SMAD3 as a transcriptional co-activator [100, 101]. Studies have shown that inhibition of β-catenin using IGC-001 inhibitor also inhibits CBP, but not p300, resulting in the downregulation of α-SMA expression in cellular wound healing models [68, 73, 100]. It would be of interest to determine if there is a feedback loop between these signaling pathways that mediates their crosstalk, or if CBP is the mediating factor that initiates the SMAD3/β-catenin complex for nuclear translocation and gene expression. A study by Liu et al. [102] showed that β-catenin negatively regulates the effect of TGF-β1 on fibroblasts by reversing their myofibroblast phenotype back to the fibroblast state. This in vitro study further showed that overexpression of β-catenin prevented the upregulation of type I and III collagen, and α-SMA in TGF-β1-treated human dermal fibroblasts [92]. This study thus suggests that these pathways regulate each other.

### Interplay between Wnt/β-catenin and Notch signaling pathways during wound healing

It is suggested that the Wnt and Notch signaling pathways are activated concomitantly during development, tissue regeneration, and wound healing, but with distinct roles [103]. Studies have shown that Notch signaling inhibits Wnt/β-catenin activity in stem cells to prevent self-renewal and promote differentiation [103, 104]. A wound healing study in mouse and human dermal fibroblasts showed that Notch signaling enhanced collagen lattice contraction while β-catenin promoted cell motility [87]. A study by Acar et al. [103] showed that NICD directly binds to β-catenin and inhibits its transcriptional activity to promote cell differentiation.

It has also been observed that some proteins are targets for the interplay between these signaling pathways (Fig. 4). A study by Blokzijl et al. [105] showed in a mouse muscle cell line that NICD interacts with SMAD3 to activate the Suppressor of Hairless, Lag-1 (CSL) transcription factor, which binds to promoter regions of Notch targets genes. SMAD3 also interacts with β-catenin to increase expression of wound healing target genes such as *α-SMA* and *fibronectin* [100]. This suggests that SMAD3 may be a downstream link for β-catenin and NICD activity during wound healing. It is unclear at what stages of wound healing these interactions occur. Furthermore, these interactions may be a potential target for the progression of wound healing from the prolonged inflammatory phase to the proliferative and remodeling phases in chronic wounds. Also, CBP/p300 has been shown to bind the MAML-1 transcription co-activator for chromatin remodeling and expression of Notch target genes [106, 107]. These studies indicate that CBP/p300 is also a downstream target for β-catenin and Notch signaling activity. Further studies thus need to be explored to understand the interaction of CBP/p300 with β-catenin and Notch signaling during wound healing.

### Modulation of Wnt/β-catenin signaling in chronic wounds

Tight regulation is critical for wound healing as dysregulation leads to delayed wound repair and the onset of chronic wounds. Chronic wounds such as pressure ulcers, venous ulcers, and DFUs are a primary result of prolonged inflammation [108]. Pressure ulcers are caused by increased pressure (> 200 mmHg) for long periods of time, and affects bedridden or immobile patients, leading to the compression of soft tissue which causes ischemia and tissue necrosis [109]. Patients with pressure ulcers present with high levels of inflammation, disruption of the ECM, and reduced growth factor secretion [109]. Venous ulcers are as a result of vascular insufficiency due to venous hypertension which leads to varicose vein formation and chronic inflammation [108, 110]. Hyperproliferation and poor differentiation of keratinocytes also contribute to venous ulcers [111]. DFUs, which are a complication of diabetes mellitus (DM), are caused by neuropathy and chronic low-grade inflammation that persists due to the hyperglycemic state in diabetic patients [112].

The common features of these chronic wounds include prolonged inflammation, recurring infection, and poor epidermal cell response to repair stimuli [2]. Upon injury, hemostasis and inflammation are activated, however, there is an increased number of neutrophils and macrophages, which secrete increased levels of pro-inflammatory cytokines such IL-1β and TNF-α, as well as reactive oxygen species (ROS) [5, 113, 114]. The elevated pro-inflammatory cytokines prevent the secretion of TGF-β1 and other growth factors that mediate transdifferentiation of M1 macrophages to reparative M2 macrophages. Moreover, M1 macrophages secrete high levels of proteases, and reduced levels of their inhibitors, which increases the degradation of the ECM and prevents progression to the proliferative and remodeling phases of wound healing [2].

Dysregulation of the canonical Wnt/β-catenin signaling pathway is implicated as one of the drivers of chronic wounds [115, 116]. A study by Stojadinovic et al. [116] showed that keloid tissue sections and keratinocytes derived from affected patients expressed high levels of β-catenin, which altered the expression of *C-MYC*, resulting in poor migration of keratinocytes and delayed wound healing. Keloids and hypertrophic scars, which are caused by excessive ECM deposition, particularly collagen, have been shown to be driven by continuous activation of the TGF-β signaling pathway [117]. Furthermore, studies have shown increased expression of TGF-β and β-catenin in keloid tissues compared to normal skin, suggesting a crosstalk of these signaling pathways in keloid disease [117, 118]. Moreover, the Notch signaling pathway has also been implicated in the pathogenesis of keloid disease. For example, a study by Syed & Bayat [119] showed that keloid tissue samples expressed high levels of the Notch-1 receptor compared to normal skin. Furthermore, this study showed an overexpression of the JAG-1 ligand in human keloid fibroblasts compared to normal cells, which indicated increased cell proliferation and migration in in vitro experimentations. The interplay of these signaling pathways in the pathogenesis and progression of hypertrophic scars and keloid disease thus requires further investigation.

In ulcerative conditions such as ulcerative colitis (UC) Wnt/β-catenin signal activation has also been reported to be increased [120]. A study by Cosin-Roger et al. [120] showed that UC patients presented with high levels of M1 macrophages in newly damage ed. intestinal mucosa, while the mucosa of chronic patients presented with high levels of M2 macrophages. Moreover, this study showed increased expression of Wnt ligands (Wnt1 and Wnt3A) in macrophages, which impairs the differentiation of enteric epithelial cells. In DFUs, Wnt/β-catenin signaling is modulated due to high expression of Wnt antagonists such as the secreted frizzled-related protein 4 (sFRP4) [121]. It is also reported that the Wnt/β-catenin signaling pathway is downregulated in diabetes due to decreased levels of R-spondin 3 (Rspo-3) [122]. The R-spondin (1–4) family of proteins are secreted ligands that have been shown to potentiate Wnt/β-catenin signaling via the LRG4 and LRG5 G-coupled receptor protein [123]. Their downregulation has been suggested to lead to the downregulation of Wnt/β-catenin in diabetic ulcers.

### Modulation of Wnt/β-catenin signaling pathway in diabetic wounds

The glycemic state in DM triggers disturbances that lead to systemic complications that alter the wound healing process. The inflammatory phase, like in pressure and venous wounds, is prolonged and leads to increased accumulation of M1 macrophages which secrete elevated levels IL-1β and TNF-α [124]. Diabetes also leads to increased myeolopoiesis in the bone marrow thereby increasing circulating monocytes that migrate to the wound site and differentiate into macrophages [125]. Furthermore, there is high expression of macrophage inflammatory protein-2 (MIP-2) and macrophage chemoattractant protein-1 (MCP-1), which increase macrophage recruitment to the wound site [124]. Increased recruitment of macrophages and neutrophils leads to increased cytokine levels, ROS, and protease production that prevents formation of granulation tissue and re-epithelialization of keratinocytes [124, 126]. High glucose levels also lead to non-enzymatic glycation of proteins forming advanced glycated end-products (AGEs), that when they bind to the AGE receptor (RAGE) lead to further hyperinflammation and increased ROS production due to high oxidative stress [124, 127, 128]. Hyperglycemia also prevents the proliferation of endothelial cells and fibroblasts in the wound area. Furthermore, fibroblasts poorly transdifferentiate into myofibroblasts, thus leading to poor angiogenesis and decreased ECM production [129]. Poor myofibroblast formation is also associated with reduced expression of TGF-β1 due to high levels of TNF-α [130]. Moreover, there is increased expression of SMAD7, which inhibits TGF-β signaling in macrophages and fibroblasts [131]. The Notch signaling pathway has also been shown to be activated in diabetic wounds, where studies have shown that Notch1 activation enhances the inflammatory response and inhibits myofibroblast formation [50, 132].

DM impairs the Wnt/β-catenin pathway [133]. It has been shown that low levels of Wnt1 and β-catenin in human diabetic wounds is negatively correlated with increased levels of pro-inflammatory cytokines such as TNF-α and IL-6, as well as high expression of caspase-3 and Bax proteins that are involved in apoptosis [134]. Also, polymorphisms in the *TCF7L2* gene increases the risk of developing diabetes as TCF7L2 regulates the expression of *ISL1*, which is required for proinsulin synthesis. Mutations on the *TCF7L2* genes lead to the dysfunction of pancreatic beta cells [133, 135]. Studies have also reported that DM causes reduced production of Wnt3A and Wnt4 ligands, which impair the function of pancreatic beta cells. Other ligands such as Wnt5A are reported to be low at the onset of type 2 diabetes mellitus (T2DM), but increase overtime and contribute to chronic low-grade inflammation [136]. Furthermore, the secretion of Wnt5A by macrophages also causes vascular endothelial dysfunction, which impairs angiogenesis [137]. DM further contributes to the downregulation of Wnt/β-catenin signaling by preventing the stabilization and nuclear translocation of β-catenin for the expression of genes (e.g. *MYC*, *CCND1,* and MMPs) that are required for the proliferation and remodeling stages of healing [1, 138]. High ROS levels in DM are suggested to also induce competitive binding of the limited β-catenin to other transcription factors, such as FOXO, instead of binding to TCF/LEF transcription factors, which alters the proliferation of pancreatic beta cells and insulin synthesis [139]. A study in a diabetic wounded mouse model also indicated that CXXC-type zinc finger protein 5 (CXXC5), a Wnt/β-catenin suppressor, is overexpressed in diabetic wounds, thereby downregulating Wnt/β-catenin activation and preventing angiogenesis during wound repair. Treatment with the KY19334 small molecule inhibited CXXC5 binding to Dvl, leading to Wnt/β-catenin activation and improved wound healing [140]. There is also increased levels of GSK-3β in DFUs, however, current molecules such as Thiazolidinediones that have inhibitory effects on GSK-3β are associated with a high risk of heart failure [141]. These studies indicate the need to identify improved therapies that will modulate chronic inflammation and activate Wnt/β-catenin signaling to improve the healing of diabetic wounds.

## Current treatments and future strategies targeting signaling pathways for healing of chronic wounds

### Standard and emerging wound care therapies

Conventional wound care strategies involve debridement, wound dressing, infection control, and pain management [142]. It was also recommended by the Wound Healing Foundation that wound care must be simplified for patients to do it themselves or easily assisted by a family member [142]. The importance of wound debridement is to remove non-viable and dead tissue [142, 143]. Infection control is also critical to prevent occurrence of drug-resistant microbial biofilm by treatment with topical antibiotics [2, 142]. Varying wound dressings which aim to manage wound moisture and pain have also been discussed. These include dressings that can deliver antimicrobial agents and debridement [142, 143]. The challenge of wound dressing is that it requires repeated application [142]. Other treatment options such as skin grafts and flaps are used for wound cover and blood supply. Negative pressure wound therapy and hyperbaric oxygen therapy, which are used to remove wound exudate and improve the formation of granulation tissue, wound perfusion, and contraction have been applied for the treatment of chronic wounds, however, the treatment cost, particularly for hyperbaric oxygen therapy is high [142, 143]. While standard wound care has shown to improve healing of chronic wounds, their effectiveness is moderate as they do not prevent the reoccurrence of chronic wounds. Other strategies such as treatment with growth factors and the use of ECM scaffolds have also been developed [1], but are moderately effective.

Emerging therapies such as stem cell therapy have entered early clinical trial stages. These include a phase I/II clinical trial investigating the safety and efficacy of allogeneic mesenchymal stem cells (MSCs) for the treatment of chronic venous ulcers [144]. In this clinical trial, dermal mesenchymal cells that express the *ATP binding cassette subfamily B member 5* (*ABCB5*) were administered to patients with venous ulcers. There was a decrease in IL-1β-mediated inflammation, as well as a shift from M1 to M2 macrophages, and a reduction in wound size in the treatment group [144, 145]. MSCs can differentiate into other cell types such as skeletal muscle, bone, and adipose tissue, but their benefit in cell therapy for wound healing is suggested to be attributed to their ability to produce biomolecules such as KGF, VEGF, and IGF that are involved in re-epithelialization and neovascularization [146]. The current limitation of MSCs for cell therapy is overcoming the microenvironment in chronic wounds, which may require repeated cell therapy to overcome the hypoxic, high ROS and high inflammatory microenvironment that may affect their survival and proliferation upon treatment. Another advancing therapeutic strategy involves the use smart bandages. A preclinical study in a mouse model showed that using a wireless, closed-loop smart bandage with multimodal sensors stimulates the proliferation of monocyte/macrophage cell populations and improves healing of cutaneous wounds [147]. The main limitation of smart bandages is the high cost for large-scale production.

### Targeting signaling pathways for treatment of chronic wounds

Current diabetic treatments include insulin injection and exercise for managing T1DM and T2DM respectively, however, the complication of non-healing wounds is still a matter to be addressed. Natural compounds such as the Chinese traditional herb *Centella asiatica* (*C. asiatica)* has been shown to promote fibroblast proliferation and ECM synthesis in wound healing. This extract of *C. asiatica* include triterpenoids, asiaticoside (AC) and madecassoside, which have been reported to promote collagen synthesis in human fibroblasts [148]. A study by Nie et al. [148] prepared a gel compound using *C. asiatica* and NO for application on diabetic cutaneous ulcers in a mouse model, and showed improved wound healing by activating the Wnt/β-catenin signaling pathway, which increased the expression of Wnt1 and β-catenin. A phase 3, randomized clinical study showed that asiaticoside extract (ON1O1) improved healing of DFUs by activating the switch from M1 to M2 macrophage phenotype [149].

### Photobiomodulation therapy activates signaling pathways for wound healing

Photobiomodulation (PBM), previously known as low-level light therapy (LLLT), which utilizes light devices such as lasers and light emitting diodes (LEDs), has been identified as a potential therapeutic modality for treating cutaneous wounds, alopecia, atopic dermatitis, and other inflammatory conditions [150–152]. This discovery was made by Mester [150], who showed that laser treatment stimulates cellular proliferation as well as hair regeneration in a wound healing mouse model. The light from PBM devices interacts with photosensitive receptors and chromophores in the mitochondria and human skin, thus inducing a photochemical action and activating cellular signals that lead to the transcription of target genes associated with wound healing [153]. Excitation of cytochrome C oxidase in the mitochondria modulates the electron transport chain, which increases the production of adenosine triphosphate (ATP) and ROS, leading to downstream activation of signaling pathways [154]. Wavelengths ranging from 420 nm to 830 nm have been shown to modulate oxidative stress and accelerate wound healing [155]. Furthermore, PBM has been shown to improve” wound” closure in an in vitro diabetic wounded model [156]. PBM has also been shown to mediate macrophage polarization from M1 to the reparative M2 macrophage at the red and near infrared spectrum (660-1000 nm), and modulate the production of cytokines such as IL-6 and TNF-α [157, 158]. Another study showed, in injured skeletal muscle of Wistar rats, that PBM decreased the number of M1 macrophages (CD68^+^) 2 days post-PBM at the wavelength of 660 nm, and increased M2 macrophages (CD163^+^ and CD206^+^) 7 days post-PBM at the wavelength of 780 nm [159]. PBM has also been shown to induce proliferation and migration of keratinocytes and fibroblasts in normal and diabetic cellular models [160, 161]. Few clinical studies have shown great promise in the effect of PBM in DFUs. For instance, a study by Mathur et al. [162] showed that the application of PBM in combination with standard DFU treatment reduced wound size after 2 weeks of treatment. Another study showed that PBM accelerated wound healing in DM patients with grade 3 burn ulcers 8 weeks after treatment [163]. Preclinical studies have shown that PBM therapy in combination with mesenchymal stem cell engraftment can accelerate wound healing in a diabetic murine model [164]. The mechanisms of action in PBM-induced wound healing include activation of signaling pathways associated with wound healing such as the TGF-β, PI3K/AKT, MAPK, and the Wnt/β-catenin pathways, to mention a few [156, 160, 165, 166].

Some studies have shown that PBM activates the Wnt/β-catenin signaling pathway in outer root sheath cells and in hair follicle stem cells. For instance, a study by Kim et al. [167] showed that PBM of human outer root sheath cells at the wavelengths of 660 nm and 830 nm increased their cell proliferation and migration. Furthermore, they showed that PBM activated both the Wnt/β-catenin and ERK/MAPK signaling pathways for proliferation and migration, which suggests that PBM can activate multiple pathways at a single wavelength and dose. Another study by Jin et al. [165] showed that PBM at the wavelength of 635 nm activates a new hair cycle in hair follicle stem cells by upregulating β-catenin gene expression in β-catenin transgenic mice. Interestingly, another study showed in a mouse model that PBM at the wavelength of 535 nm and power density ranging from 0.1 W/cm^2^ to 0.5 W/cm^2^ induced transcriptional activation of genes associated with Wnt/β-catenin, Notch, TGF-β, and the JAK/STAT signaling pathways [168]. These studies thus indicate that PBM can induce activation of multiple signaling pathways in cutaneous tissue. It is unclear however, if PBM activates multiple signaling pathways simultaneously, or whether there is co-activation of these signaling pathways.

While this therapeutic approach has demonstrated positive preclinical findings, some variations in the experimental and clinical parameters have also been reported. These include variations in the wavelength, radiation exposure (fluence), and irradiance. Some studies have reported variations in the effect of PBM in cell proliferation and wound healing when using the same parameters as previous studies [152, 169]. Also, studies have indicated that tissues with high mitochondrial content (e.g. muscle, brain, and heart) require low light dosage compared to tissues with low mitochondria (e.g. skin, tendon, and cartilage), which require a higher light dosage [152, 153]. Furthermore, it is suggested that different fibroblast subtypes in the skin respond differently to PBM due to the heterogeneity of these cellular subtypes [169].

## Conclusion

The role of Wnt/β-catenin signaling in embryonic development is well known. But its effect in disease progression is still under investigation. We have shown in this review the importance of this pathway in wound healing. Furthermore, we have highlighted the effects of its dysregulation in chronic wounds, including diabetic ulcers. Moreover, we also discussed its crosstalk with the TGF-β and Notch signaling pathways, which is critical for wound healing. We thus recommend that future therapies investigate strategies that induce the (re)activation of the Wnt/β-catenin signaling pathway, especially for treatment of chronic ulcers that remain persistently in the inflammatory phase of healing. PBM remains one of the promising non-invasive therapeutic strategies that has the potential to improve the healing of chronic wounds via activation of the Wnt/β-catenin signaling pathway, as well as other signaling pathways critical for wound healing. Moreover, PBM can activate skin stem cells, as well as epithelial cells to augment the healing of chronic wounds. Future studies will need to further investigate optimal parameters for the clinical application of PBM therapy in different chronic wounds, and to determine if PBM is most effective alone or in combination with standard or other emerging therapies.

## Data Availability

No datasets were generated or analysed during the current study.
